# Further Account of Dr. W. Currie's Observations on the Causes and Cure of Remitting Bilious Fevers

**Published:** 1799-05

**Authors:** 


### *Further Account of Dr.* W. Currie's *Observations on the Causes and Cure of Remitting Bilious Fevers.*

IN the 'Critical Retrospect,' of our first Number, p. 105, we gave only the general result of Dr. Currie's inquiry into the nature and origin of the bilious and yellow fevers: in this place, we propose to gratify our readers with some leading particulars of that interesting publication.

The nature of the work, and the design of the author, are set forth in his introduction, in these terms:—

" The following production (the result of much reading, reflection, and considerble considerable experience,) contains observations on the situations, climates and seasons, in which *remitting* or *bilious fevers*, are most prevalent; the causes from which they originate; the circumstances which render them epidemic; remarks on Sydenham's doctrine on the influence of constitutions, or conditions of the atmosphere; an examination of the question, whether intermittents or remittents are contagious or not? and a comparison of their distinguishing symptoms with those of the contagious fever, commonly called the yellow fever, which has occasioned so much mortality and distress, in different sea-port towns of the United States of America, since the year 1793; with a description of the remitting fever, as it appears in Philadelphia: and the method of treatment which the author has experienced to be most successful.

"An abstract is also annexed of the opinions and observations of almost all the physicians that have practised in different ages, and in different climates, which have come to the author's knowledge, that he thinks worthy of notice; the object of which is to furnish those at a distance from public libraries with a compendious and connected view of every thing interesting that has been published, and that lies scattered in a multitude of volumes on the subject; free from the perversions of fallacious and misleading theory, or the misrepresentations of uncharitable and distorting party-spirit."

Under the title of *Observations on Bilious Fevers*, the author, after defining the term bilious fever, proceeds to enumerate various countries and situations, in different parts of the world, which are subject to this disease; which he very justly remarks, "is amazingly influenced in its aspect and symptoms, by the soil, situation, climate, season, and by the preceding and present qualities of the atmosphere, and the customary mode of living of the inhabitants."

The principal part of Dr. Currie's publication consists of '*An Abstract of the Opinions and Practice in febrile diseases of Physicians of different countries*,' &c. with occasional remarks, preceded by Dr. Buel's Account of the Sheffield fevers of 1793, 1794, and 1795 (from Webster's Collection), and two letters to the author from Dr. Johnson, of Talbot County, Pensylvania, and Drs. Taylor and Hansford, of Norfolk—extending in the whole from the 79th to the 206th page inclusive. In this abstract, after a hasty sketch of the opinions and practice of several of the ancient, and of the earlier modern physicians, the author proceeds to display, in their own words, generally, those of *Hoffman*, *Pringle*, *Cleghorn*, *Huxham*, *Morgagni*, *Tissot*, *De Monchy*, *Lind*, *Badenoch*, *Rush*, *Baker*, *Cullen*, *Blane*, *Mosely*, *R. Jackson*, *G. Fordyce*, *Paterson*, and *Wade*, at various length; and the conclusion which he derives from this copious survey of the writings of others, is, "that the remitting, or bilious fever, as it is commonly called, is only a variety of the intermitting fever, occasioned by an invisible invisible matter (known to exist only from its effects), derived from dead animal and vegetable matter, in a state of putrefaction; that it is distinguished by an evident remission or abatement, but not a total suspension or cessation, of all the febrile symptoms, once in the course of every twenty-four hours, most commonly in the morning, and a renewal and increase of the same before the evening, differing in this, as well as other circumstances, from the typhus, or continued fever, occasioned by human contagion, in which there is almost always an exacerbation, or increase of the fever later in the evening; that the nearer it approaches to, or resembles, the intermitting type, the greater is the prospect of safety; that the paroxysms are prolonged, and intermissions prevented, or rendered imperfect, by two opposite circumstances, viz. by a *phlogistic* diathesis, and by preternatural *depression of strength;* that this kindof fever is not contagious, or communicated from one person to another: and that it differs from the malignant yellow fever, not only in that respect, but in its causes, nature, and symptoms, as well as in the remedies requisite for its cure." And finally, " that no general or infallible rule can be established, with regard to blood-letting in remitting fevers, derived from marsh-miasmata." Indeed it is observable, that of all the late writers quoted by Dr. Currie, Dr. Moseley is the only one who decidedly recommends venesection.

To this 'Abstract,' Dr. Currie has prefixed a "*Description of the Bilious Remitting Fever as it usually appears in Philadelphia, in summer and autumn,*" drawn up with equal perspicuity and precision. His remarks on the predisponent causes, prognosis, and on the weather, are valuable. The Doctor's plan of cure consists in removing the patient to a dry situation and pure air, after the remote causes of the disease have been subdued; and in procuring such an intermission of the symptoms, as shall admit of the administration of the Peruvian bark. To this end, bleeding is recommended; an operation which our author has sometimes found it requisite to repeat (from ten to twelve ounces) every day, to a fifth time; and in a few cases he has opened a vein, twice a day. Calomel is particularly preferred, upon the authority of Drs. *Balfour*, *Clarke*, *Wade*, *Chisholm*, and others, as a purgative, and, joined with opium, to take off the irritability of the stomach; sweating, by means of antimonials, is deemed necessary to procure an intermission; and the cold bath, upon the credit of Dr. *R. Jackson*, when the stomach rejects the bark—which the author advises to be given by injection into the bowels, as well as by the mouth. To reduce the dropsical swellings which sometimes succeed, ten or fifteen grains of potash, two or three times a day, in some bitter draught, are directed; in more troublesome cases, the digitalis, squills alternated with chalybeates, mercurial frictions," &c. &c.

